# Implementation costs of screening and brief intervention for illicit drug use

**DOI:** 10.1186/1940-0640-8-S1-A88

**Published:** 2013-09-04

**Authors:** Gary Zarkin, Jeremy Bray, Jesse Hinde, Richard Saitz

**Affiliations:** 1 RTI International, Research Triangle Park, NC, USA; 2 University of North Carolina-Greensboro, Greensboro, NC, USA; 3 Boston University Medical Center, Boston University, Boston, MA, USA

## 

In 2008, a World Health Organization multi-national clinical trial found screening and brief intervention (SBI) to be effective in reducing illicit drug use and many have called for its widespread dissemination. To gain policy support for widespread dissemination, however, a strong business case must be made to policymakers. In particular, policymaker concerns over implementation costs can act as a significant obstacle to widespread dissemination. This presentation estimates the various cost components of providing SBI to reduce illicit drug use in a primary care setting as part of the clinical trial *Assessing Screening Plus brief Intervention’s Resulting Efficacy* (ASPIRE). An activity-based micro-costing approach was used to determine implementation costs and set-up costs. Implementation costs include variable costs (direct costs that directly vary by the number of services provided) and quasi-fixed costs (service-support costs that indirectly vary by the number of services provided). Set-up costs are fixed costs incurred to the program, such as the purchase of durable equipment and initial training. A taxonomy of activities and resource inputs (labor, material and space) were identified. Quantity and most price values were obtained through primary data collection during the trial. Regional price estimates for wages and space were used where needed. Preliminary estimates indicate that variable costs to provide SBI for unhealthy drug use are similar to SBI programs focusing on alcohol. Quasi-fixed costs are substantially higher than variable costs for this program. Quasi-fixed costs are not usually estimated; the focus is usually on variable costs of service delivery. This study fills a gap in the economic literature on SBI regarding quasi-fixed costs. These findings have important implications for the adoption and sustainability of SBI programs, where focusing only on variable costs underestimates the fiscal impact of implementing SBI.

